# Mental wellbeing in the German old age population largely unaltered during COVID-19 lockdown: results of a representative survey

**DOI:** 10.1186/s12877-020-01889-x

**Published:** 2020-11-23

**Authors:** Susanne Röhr, Ulrich Reininghaus, Steffi G. Riedel-Heller

**Affiliations:** 1 Institute of Social Medicine, Occupational Health and Public Health (ISAP), Medical Faculty, University of Leipzig, Leipzig, Germany; 2 Global Brain Health Institute (GBHI), Trinity College Dublin, Dublin, Ireland; 3 Department of Public Mental Health, Central Institute of Mental Health, Medical Faculty Mannheim, Heidelberg University, Mannheim, Germany; 4 Centre for Epidemiology and Public Health, Health Service and Population Research Department, Institute of Psychiatry, Psychology & Neuroscience, King’s College London, London, UK; 5 ESRC Centre for Society and Mental Health, King’s College London, London, UK

**Keywords:** COVID-19 lockdown, COVID-19 pandemic, Mental health, Mental wellbeing, Old age, Epidemiology, Survey

## Abstract

**Background:**

Older individuals are at increased risk of a severe and lethal course of COVID-19. They have typically been advised to practice particularly restrictive social distancing (‘cocooning’), which has sparked much debate on the consequences for their mental wellbeing. We aimed to provide evidence by conducting a representative survey among the German old population during COVID-19 lockdown.

**Methods:**

A computer-assisted standardized telephone interview was conducted in a randomly selected and representative sample of the German old age population (*n* = 1005; age ≥ 65 years) during the first lockdown in April 2020. Assessments included sociodemographic factors, aspects of the personal life situation during lockdown, attitudes towards COVID-19, and standardized screening measures on depression, anxiety, somatization, overall psychological distress (Brief Symptom Inventory/BSI-18) and loneliness (UCLA 3-item loneliness scale). Sampling-weighted descriptive statistics and multiple multivariable regression analyses were conducted.

**Results:**

Participants were *M* = 75.5 (*SD* = 7.1) years old; 56.3% were women. At data collection, COVID-19 lockdown had been in force for *M* = 28.0 (*SD* = 4.8) days. Overall, older individuals were worried about COVID-19, but supportive of the lockdown. Mean BSI-18 scores were 1.4 for depression, 1.6 for anxiety and 2.2 for somatization as well as 5.1 for global psychological distress. These figures did not indicate worse mental wellbeing, given normative values established by studies before the pandemic (2.0, 1.6, 2.4, 6.0, respectively). The prevalence of loneliness was 13.1%, which also fell within a range of estimates reported by studies before the pandemic. There were only few significant associations of aspects of the personal life situation during lockdown and attitudes towards COVID-19 with mental wellbeing. Resilience explained a large amount of variance.

**Conclusions:**

In the short-term, the mental wellbeing of the German old age population was largely unaltered during COVID-19 lockdown, suggesting resilience against the challenging pandemic situation. Our results refute common ageist stereotypes of “the weak and vulnerable older adults” that were present during the pandemic. Long-term observations are needed to provide robust evidence.

**Supplementary Information:**

The online version contains supplementary material available at 10.1186/s12877-020-01889-x.

## Background

In 2020, the majority of the world’s population is experiencing unprecedented restrictions to their lifestyles due to mass quarantine measures imposed to slow the spread of the newly emerged coronavirus Severe Acute Respiratory Syndrome Coronavirus 2 (SARS-CoV-2), causing the respiratory disease Corona Virus Disease 2019 (COVID-19) [1]. Germany was among the early affected countries with the first COVID-19 case being reported on January, 28th 2020, followed by a rapid increase of infections. Nationwide comprehensive contact restrictions and lockdowns became effective on March 21st/22nd, 2020. By end of May 2020, 181,482 COVID-19 cases and 8500 deaths (4.7%) were recorded [2].

Early on, the World Health Organization (WHO) published a statement on psychosocial considerations during the COVID-19 outbreak, raising awareness about the potential psychological impact of mass quarantine measures [3]. Evidence from previous serious coronavirus outbreaks, e.g. the SARS pandemic 2003/2004, showed negative psychosocial health consequences of mass quarantine measures, including anxiety, depressive symptoms, social isolation, loneliness, and a lack of social support [4]. A recent study assessing the mental health impact of COVID-19 lockdowns in adults across 12 heavily affected countries (incl. USA, Spain, Italy, France, Germany, UK, Iran, Turkey, and Switzerland) found, that average scores on psychological disturbance, posttraumatic stress disorder (PTSD), and depression exceeded mild-risk thresholds [5]. Notably, the authors reported that higher age was associated with lower psychological distress, suggesting that older individuals better adapt to challenging life events. However, the results of this study relied on a non-random, selected sample based on voluntary participation in an online survey; with the proportion of older participants being rather low. Moreover, the authors acknowledged that findings with regard to age could be related to the assessment procedures, which allowed individuals above 60 years of age assistance in completing the online survey, potentially biasing response behavior. Although other studies on mental wellbeing during COVID-19 lockdown in Germany likewise suggested that compared to younger adults, older individuals may have been less mentally affected, all of these studies built on convenience sampling methods from online surveys, with stark underrepresentation of older age [6–8].

Focusing in particular on the mental wellbeing of older adults during the COVID-19 pandemic is important for several reasons. As noted earlier, older individuals and individuals with pre-existing health conditions (most of them highly prevalent in older age, including diabetes and hypertension), constitute a vulnerable high-risk group for a severe and lethal course of COVID-19. By the end of May 2020, individuals above 70 years of age accounted for 86% of all deaths due to COVID-19 in Germany [2]. Therefore, the government advised older individuals to practice particularly restrictive social distancing (“cocooning”), e.g. avoiding going outside, avoiding having direct contact with grandchildren or buying groceries themselves. This, as well as potential worry or anxiety about contracting COVID-19, may lead to social withdrawal and isolation, which are associated with adverse health outcomes and increased mortality [9]. Reduced social participation may also result in cognitive and functional decline [10]. Then again, older individuals may have higher resilience and therefore may cope better with adverse life events because of having mastered crises throughout life [11]. First studies investigating coping with the pandemic – though again not focusing on older individuals – reported that proactive coping behaviors, such as social activities (e.g. sharing worries with others, using social media to keep in touch with others), a healthy diet, physical activity, keeping a routine and pursuing hobbies were associated with better mental wellbeing [12–14].

Moreover, despite older individuals being at higher risk of a severe course of COVID-19, it is important not to victimize a population group merely based on age. On a societal level, COVID-19 has sparked much controversial debate on how older individuals are presented, as stereotypes with regard to age can accentuate the exclusion of, and prejudice against, older adults, which in turn may complicate dealing with the COVID-19 crisis for older people [15]. Against this background, we aimed to investigate the mental wellbeing in the old age population during COVID-19 lockdown in Germany. Moreover, we aimed to inspect associations of mental wellbeing with sociodemographic factors, aspects of the personal life situation during lockdown and attitudes towards COVID-19 as well as resilience.

## Methods

### Study design and sample

The survey was conducted as a computer-assisted telephone interview by USUMA, a leading social research institute in Germany. The target sample size was 1000 individuals at least 65 years old. Sampling was based on multi-stage random digital dialing, drawing from the Association of German Market and Social Research Agency’s (ADM) sample base that includes registered and non-registered telephone numbers. Telephone numbers were drawn proportionally to the German population structure and regionally stratified according to district sizes throughout Germany. This would ensure a random selection of households. Within households, the target person to be interviewed was also randomly selected if there was more than one individual being 65 years and older, applying the Kish-Selection-Grid [16]. Interviewers were research assistants, who were employed by USUMA (various backgrounds) and who were trained to conduct interviews on health-related topics. They were randomly monitored for quality control. Data were collected from April 6th to April 25th, 2020, when nationwide COVID-19 lockdowns were continuously in force.

### Weighting procedures for sample representativeness

Data were iteratively weighted by age, sex and all regions across Germany using official population statistics by the Federal Statistical Office to allow for representativeness of the population [17]. Therefore, first, a design weighting was undertaken, which used household transformation to convert the initially selected telephone numbers sample into a target population representative sample. Basically, this transformation compensated for a larger probability that a certain target person stems from a one-person household than a multi-person household, when calling a household (which is necessary regarding the age group at target). Therefore, individuals from households of smaller size were weighted lower than individuals from households with more than one possible target person. The weighting factor was such inversely proportional to the probability of selection in the household.

Second, an adjustment weighting was conducted to correct for population deviations caused by calling failures (e.g. nonresponse). Age, sex and regional distributions of the sample were considered. To determine the target figures, the data available from the statistical offices were used. This procedure led to sample specific weighting factors that were applied during analysis.

### Measurements

#### Sociodemographic variables

The structured telephone interview consisted of three parts (for full interview see supplemental material). First, sociodemographic data were collected, which comprised standardized questions on age (years), sex (self-report; female/male/other), education (low/middle/high), marital status (married, single, divorced, widowed) and living situation (alone, with partner/spouse, with relatives others than partner/spouse, with others).

#### COVID-19 related variables

Second, participants were asked eleven questions in direct relation to the COVID-19 pandemic, comprising attitudes to and compliance with mass quarantine measures, exposure to COVID-19 and aspects of the personal life situation. Six 5-point Likert-scale items („totally disagree “to „totally agree“) were used to assess attitudes to and compliance with mass quarantine measures, including the extent of being worried about COVID-19, perceived threat by COVID-19, perceived threat by COVID-19 due to age, perceived threat by COVID-19 due to pre-existing health conditions, support of the governmental measures to curb the virus spread, and perceived restriction by the governmental measures. Regarding exposure to COVID-19, we asked participants two questions on whether they or someone they knew were infected and/or in self-isolation due to exposure to the virus. Aspects of the personal life situation comprised the following four questions: (1) Frequencies of direct and (2) indirect contact with individuals outside of the household over the past week were assessed, respectively („no contact at all “to „several times a day“), and we asked (3) whether participants received support in everyday tasks and (4) whether access to medical health care services was unaltered (yes/no/partially).

#### Outcome measures for mental wellbeing

The third part of the interview comprised short standardized screening instruments for mental wellbeing. We provide a description of each instrument below.

The Brief Symptom Inventory (BSI-18) was used to assess symptomatology regarding depression, anxiety and somatization utilizing six items for each outcome. Combining all 18 items of the BSI-18 provided an additional indicator of overall psychological distress, referred to as the Global Severity Index (GSI) [18]. Questions applied to the past week and were answered using a 5-point Likert-scale („not at all “to „very much“). Results are presented as mean scores. Good evidence has been reported on the psychometric properties of the German version of the BSI-18 [18].

To measure loneliness, we applied the 3-item version of the University of California, Los Angeles Loneliness Scale (UCLA-3) [19]. The questions elicited information on the subjective perception of social isolation („often“, „sometimes“, „seldom “and „never“; scored 0 to 3). Scores were summed; a score ≥ 6 indicates loneliness. The UCLA-3 is a reliable and valid measure for loneliness, specifically in telephone interviews [20].

We measured resilience, i.e. the capability to rally from stress, using the Brief Resilience Scale (BRS) [21]. The BRS consists of 6 items that are either positively or negatively worded, reducing response bias in relation to social desirability. Answers were recorded on a 5-point Likert-scale („totally disagree “to „totally agree“). The mean score of all item responses was used to quantify resilience (range: 1–5). A higher mean score indicated higher resilience. It was classified in 1.00–2.99 = low resilience, 3.00–4.30 = normal resilience, 4.31–5.00 = high resilience. The validated German adaptation was used [22].

### Statistical analysis

The sample was weighted to account for differential sampling probabilities based on age, sex and regions across Germany using 2019 census data. T-tests and Chi-square tests were used to inspect whether socio-demographic characteristics, aspects of the personal life situation during lockdown, attitudes towards COVID-19 as well as variables of mental wellbeing differed by sex. Subsequently, multiple multivariate regression models were fitted to examine associations between sociodemographic factors, aspects of the personal life situation during lockdown, attitudes towards COVID-19 and resilience, on the one hand, and mental wellbeing variables, on the other. We used continuous sum or mean scores of mental wellbeing outcomes, as appropriate, thus applying linear regression models. Adjusted models included continuous independent variables, except for: sex (female in reference to male), education (categorized according to the Comparative Analysis of Social Mobility in Industrial Nations/CASMIN classification; low, middle in reference to high [23]), living alone (living alone in reference to living with partner or others), receiving support in everyday tasks over the past weeks (no, partially in reference to yes), unchanged access of health services (no, partially in reference to yes), COVID-19 infection (self, household/family member in reference to no infection in direct social network), self-isolation due to SARS-CoV-2 exposure (self, household/family member in reference to no exposure in direct social network), being supportive of the government’s quarantine measures (due to invariance in response to the 5-point Likert-scale responses were collapsed into a dichotomous outcome: no in reference to yes). We furthermore repeated all models without adjusting for resilience in order to inspect the potential impact of this covariate. We report standardized beta (*β*) coefficients to allow for direct comparisons between dependent variables as well as between outcomes. Analyses were conducted using STATA 16.0 SE (College Station, Texas, USA), assuming a statistical significance level of *p* ≤ .05.

## Results

### Sample

The sample consisted of 1005 individuals aged 65 years and older. Initially, 1863 individuals were randomly selected. Of these, 10.7% (*n* = 200) refused to participate and 35.3% (*n* = 658) of the selected households could not be reached, leading to a response rate of 54.0%.

Participants were on average 75.5 (*SD* = 7.1; range = 65–94) years old; 56.3% were women. Compared to men, women were slightly older (*M* = 74.8, *SD* = 6.8 vs. *M* = 76.0, *SD* = 7.3; *t* (1) = − 2.67, *p* = .008), less educated (high education: 49.2% vs. 26.1%; *p* < .001), less often married (71.5% vs. 43.9%; *Χ*^*2*^(2) = 59.44, *p* < .001) and more often living alone (25.9% vs. 40.5%; *Χ*^*2*^(1) = 23.36, *p* < .001) (Table 1).

**Table 1:** Sociodemographic characteristics of the study sample (*n* = 1005)

	Total	Women (***n*** = 566)	Men (***n*** = 439)	Group difference (***p***-value)
Age; *M*, *SD*, range	75.50 (7.11; 65–94)	76.03 (7.31)	74.82 (6.78)	.008
Education; *n*, %				< .001
Low	279 (27.7)	170 (30.6)	109 (24.9)	
Middle	352 (35.1)	240 (43.2)	113 (25.9)	
High	360 (35.9)	145 (26.1)	215 (49.2)	
Marital status; *n*, %				<.001
Married	560 (55.7)	247 (43.9)	313 (71.5)	
Single	78 (7.8)	42 (7.5)	36 (8.2)	
Divorced	100 (9.9)	70 (12.4)	30 (6.8)	
Widowed	264 (26.2)	204 (36.2)	59 (13.5)	
Living situation; *n*, %				< .001
Living alone	341 (33.9)	227 (40.5)	114 (25.9)	
Living with partner	514 (51.2)	243 (43.3)	271 (61.6)	
Living with others	146 (14.5)	91 (16.2)	55 (12.5)	

Missing values: education: *n* = 13 (1.3%); marital status: *n* = 4 (0.4%); living situation: *n* = 4 (0.4%)

### Aspects of the personal life situation during COVID-19 lockdown

At the time of data collection, COVID-19 lockdown had continuously been in force for an average of 28 days (*SD* = 4.8). Two study participants (0.2%) reported having been infected with COVID-19 and less than every fifth respondent reported knowing about a case in their close or extended social network (*n* = 174; 17.2%). Self-isolation due to potential exposure to COVID-19 was reported by 12 participants (1.2%), while they knew of 157 (15.3%) individuals in their close or extended social network who had to self-isolate. As for frequency of social contact, 42.7% (*n* = 430) of the participants did not have any direct contact with others outside of their household during lockdown; however, more than half of the respondents (*n* = 507, 50.4%) reported having had indirect contact with persons outside their household every day or several times a day. The majority received at least partial support in carrying out everyday tasks (e.g. buying groceries) (*n* = 896; 90.0%) and was at least partially able to access health care services as usual (*n* = 823; 81.9%). More results are detailed in Table 2.

**Table 2:** Aspects of the personal life situation and attitudes towards COVID-19 during lockdown in the old age population in Germany (*n* = 1005)

	Total	Women (***n*** = 566)	Men (***n*** = 439)	Group difference (***p***-value)
Duration of quarantine measures; days *M* (*SD*; range)	27.96 (4.77, 15–34)	27.76 (4.89)	28.21 (4.60)	.144
COVID-19 infection; *n* (%)				.024
Self	2 (0.2)	2 (0.4)	0 (0.0)	
Household members/family	17 (1.7)	7 (1.2)	10 (2.3)	
Friends or neighbors	44 (4.4)	17 (3.0)	27 (6.1)	
Others	113 (11.2)	57 (10.1)	55 (12.5)	
Isolation due to COVID-19 exposure; *n* (%)				.168
Self	12 (1.2)	6 (1.1)	6 (1.4)	
Household members/family	35 (3.4)	26 (4.6)	9 (2.1)	
Friends or neighbors	52 (5.2)	23 (4.1)	29 (6.6)	
Others	67 (6.7)	33 (5.8)	35 (8.0)	
Frequency of direct contact with others over past week; *n* (%)				.458
Not at all	430 (42.7)	251 (44.3)	179 (40.7)	
1–2 days	292 (29.1)	166 (29.3)	126 (28.6)	
3–4 days	144 (14.4)	77 (13.6)	68 (15.5)	
5–6 days	40 (3.9)	21 (3.7)	19 (4.3)	
Everyday	66 (6.6)	34 (6.0)	32 (7.3)	
Several times everyday	30 (3.0)	14 (2.5)	16 (3.6)	
Frequency of indirect contact with others over past week; *n* (%)				.009
Not at all	71 (7.0)	35 (6.2)	36 (8.2)	
1–2 days	130 (12.9)	57 (10.1)	73 (16.6)	
3–4 days	220 (21.9)	120 (21.2)	100 (22.7)	
5–6 days	76 (7.5)	40 (7.1)	35 (8.0)	
Everyday	340 (33.8)	208 (36.8)	133 (30.2)	
Several times everyday	167 (16.6)	103 (18.2)	63 (14.3)	
Receiving support in daily activities; *n* (%)				< .001
Yes	284 (28.2)	188 (33.2)	96 (21.9)	
Partially	612 (60.8)	78 (13.8)	31 (7.1)	
No	110 (10.9)	300 (53.0)	312 (71.1)	
Unchanged health services access; *n* (%)YesPartiallyNo	607 (60.4)216 (21.5)162 (16.1)	316 (55.8)99 (17.5)138 (24.4)	291 (66.1)63 (14.3)79 (18.0)	.010
**Attitudes towards COVID-19**				
Being worried about COVID-19; *n* (%)				.329
Strongly disagree	110 (11.0)	57 (10.1)	53 (12.1)	
Disagree	129 (12.9)	68 (12.0)	62 (14.1)	
Neither agree nor disagree	226 (22.5)	130 (23.0)	96 (21.9)	
Agree	175 (17.4)	92 (16.3)	82 (18.7)	
Strongly agree	346 (36.2)	218 (38.6)	146 (33.3)	
Perceived threat by COVID-19; *n* (%)				.210
Strongly disagree	148 (14.7)	84 (14.9)	64 (14.5)	
Disagree	207 (20.6)	1221 (21.4)	85 (19.3)	
Neither agree nor disagree	289 (28.8)	165 (29.2)	125 (28.4)	
Agree	127 (12.6)	59 (10.4)	68 (15.5)	
Strongly agree	233 (23.2)	136 (24.1)	98 (22.3)	
Perceived threat by COVID-19 due to age; *n* (%)				.008
Strongly disagree	90 (9.0)	50 (8.9)	41 (9.4)	
Disagree	135 (13.5)	79 (14.1)	56 (12.9)	
Neither agree nor disagree	223 (22.2)	125 (22.3)	97 (22.3)	
Agree	156 (15.5)	68 (12.1)	88 (20.2)	
Strongly agree	391 (38.9)	238 (42.5)	153 (35.2)	
Perceived threat by COVID-19 due to pre-existing health conditions; *n* (%)				.870
Strongly disagree	287 (28.6)	157 (27.9)	130 (29.6)	
Disagree	164 (16.3)	91 (16.2)	73 (16.6)	
Neither agree nor disagree	179 (17.9)	107 (19.0)	72 (16.4)	
Agree	91 (9.1)	51 (9.1)	41 (9.3)	
Strongly agree	279 (27.8)	157 (27.9)	123 (28.0)	
Being supportive of the government’s quarantine measures; *n* (%)				.733
Strongly disagree	8 (0.8)	5 (0.9)	3 (0.7)	
Disagree	9 (0.9)	5 (0.9)	4 (0.9)	
Neither agree nor disagree	81 (8.0)	42 (7.4)	39 (8.9)	
Agree	119 (11.8)	62 (11.0)	57 (13.0)	
Strongly agree	784 (78.0)	450 (79.8)	334 (76.4)	
Feeling restricted due to quarantine measures; *n* (%)				.084
Strongly disagree	202 (20.1)	123 (21.8)	79 (18.0)	
Disagree	214 (21.3)	106 (18.8)	108 (24.7)	
Neither agree nor disagree	310 (30.8)	183 (32.4)	127 (29.0)	
Agree	125 (12.5)	64 (11.3)	61 (13.9)	
Strongly agree	152 (15.1)	88 (15.6)	63 (14.4)	

Missings: direct contact frequency: *n* = 3 (0.3%); indirect contact frequency: *n* = 2 (0.2%); unchanged medical services use: 20 (2.0%); worry about COVID-19: *n* = 1 (0.1%); threat by COVID-19: *n* = 1 (0.1%); threat due to age: *n* = 11 (1.1%); threat due to pre-existing health conditions: *n* = 4 (0.4%); supportive of lockdown: *n* = 3 (0.3%); perceived restriction: *n* = 2 (0.2%)

### Attitudes towards COVID-19 and associated measures

**Fig. 1:**
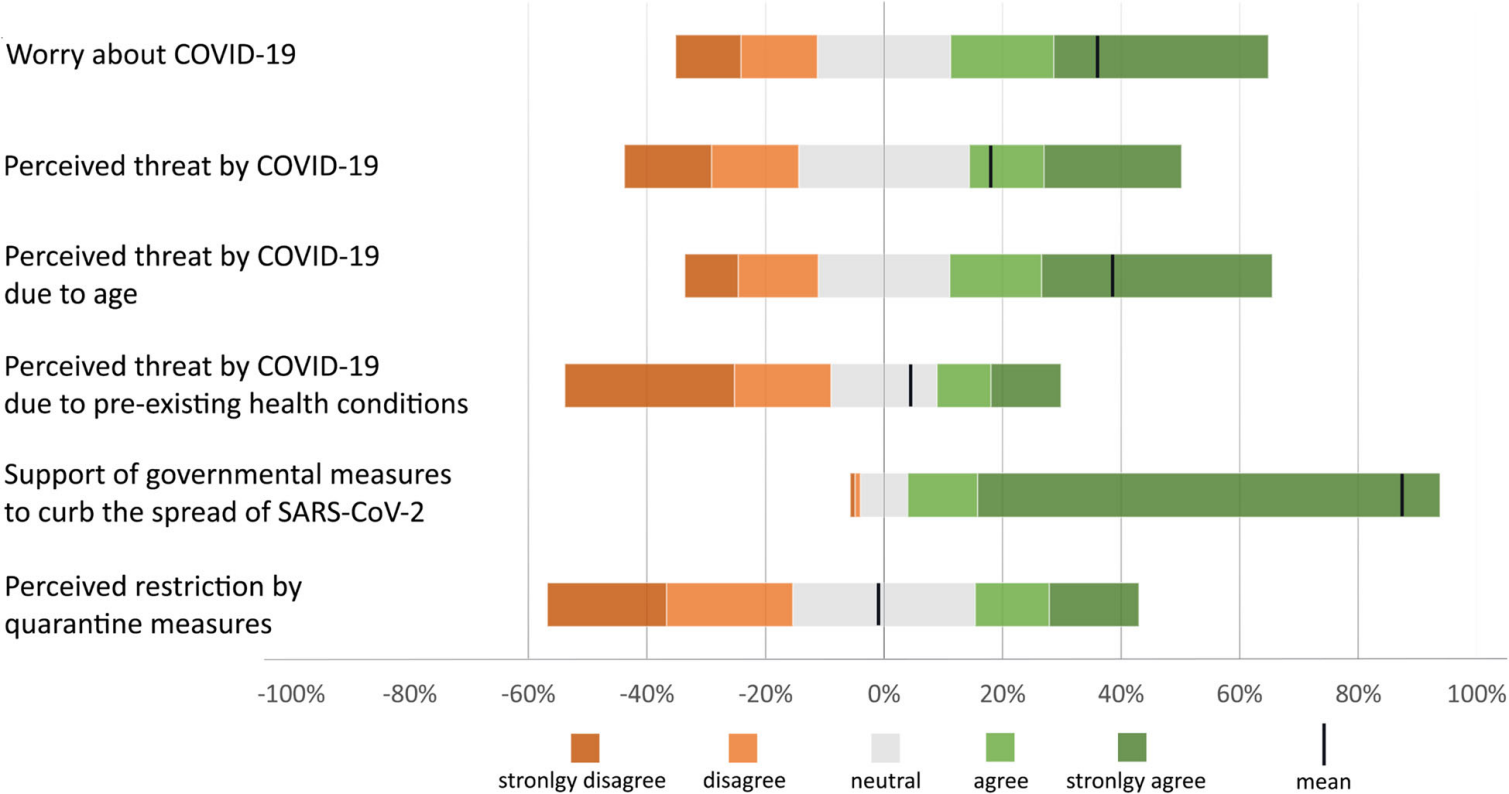
Attitudes of the old age population (representative sample: 65+ years; n = 1005) in Germany in regard to COVID-19 and quarantine measures during COVID-19 lockdown in April 2020

More than half of the respondents (*n* = 521; 53.6%) stated to worry about COVID-19, with one third of all participants doing so strongly (*n* = 346; 36.2%). About one-third (*n* = 360; 35.8%) stated to feel threatened by COVID-19, about one quarter of them to feel so strongly (*n* = 233; 23.2%). A little over half of the respondents stated that the perceived threat was due to their advanced age (*n* = 547; 54.4%), with 391 (38.9%) agreeing strongly with this statement. About one-third felt threatened due to pre-existing health conditions (*n* = 370; 36.9%), 279 (27.8%) felt so strongly. The vast majority (*n* = 903; 89.8%) was supportive of the governmental quarantine measures, with most participants agreeing strongly with them (*n* = 784; 78.0%). Over a quarter felt restricted by the quarantine measures (*n* = 277; 27.6%), 152 (15.1%) of them strongly. Roughly 40% (*n* = 416) did not feel restricted during lockdown. There were no significant sex differences in regard to attitudes to COVID-19, except for women feeling a significantly greater threat by COVID-19 due to their age compared to men (42.5% vs. 35.2%; *Χ*^*2*^(4) = 13.90, *p* = .008). Figure 1 illustrates the response distributions in regard to attitudes. Further results are detailed in Table 2.

### Mental wellbeing during COVID-19 lockdown

Table 3 provides mean scores and prevalence estimates of the mental wellbeing of older individuals during COVID-19 lockdown. Women had significantly higher scores and a higher prevalence in all mental wellbeing outcomes than men. Two-thirds (*n* = 645; 67.5%) of all participants reported moderate resilience, 13.8% (*n* = 132) had low resilience and 18.6% (*n* = 178) high resilience. Overall, women had slightly lower resilience compared to men (*M* = 3.5, *SD* = 0.7 vs. *M* = 3.7, *SD* = 0.7; *t* (1) = 3.69, *p* < .001).

**Table 3:** Mental wellbeing and resilience in the old age population during COVID-19 lockdown in Germany (*n* = 1005)

	Total	Women(***n*** = 566)	Men(***n*** = 439)	Group difference (***p***-value)
Depression; *M* (*SD*)	1.38 (1.97)	1.53 (2.11)	1.19 (1.76)	.007
Anxiety; *M* (*SD*)	1.60 (1.98)	1.75 (2.14)	1.41 (1.75)	.007
Somatization; *M* (*SD*)	2.16 (2.77)	2.32 (2.92)	1.95 (2.53)	.036
Global severity index (GSI)^a^; *M* (*SD*)	5.13 (5.49)	5.59 (5.87)	4.55 (4.89)	.003
Loneliness				
*M* (*SD*)	4.13 (1.36)	4.30 (1.48)	3.91 (1.17)	<.001
*n* (%)	130 (13.1)	92 (16.4)	38 (8.8)	<.001
Resilience				
*M* (*SD*)	3.58 (0.68)	3.51 (0.67)	3.67 (0.68)	<.001.025
High; *n* (%)	178 (18.6)	90 (16.79)	88 (21.1)	
Moderate; *n* (%)	645 (67.5)	361 (67.1)	284 (68.1)	
Low; *n* (%)	132 (13.8)	87 (16.2)	45 (10.8)	

^a^sum of depression, anxiety and somatization; Abbreviations: *M* mean; *SD* standard deviation; missings: depression: *n* = 14 (1.4%); anxiety: *n* = 17 (1.7%); somatization: *n* = 7 (0.7%); GSI: *n* = 30 (3.0%); loneliness: *n* = 11 (1.1%); resilience: *n* = 51 (5.0%)

### Associations of sociodemographic factors and mental wellbeing during COVID-19 lockdown

Results of this and all following sections are detailed in Table 4. Higher age was associated with increased somatization (*β* = .16; *p* = .002) and overall psychological distress (GSI; *β* = .10; *p* = .047). Loneliness (*β* = .11; *p* = .004) was more pronounced in women than in men.

**Table 4:** Associations of sociodemographic factors, aspects of the personal life situation, attitudes towards COVID-19 and resilience with mental wellbeing in the German old age population (n = 1005; age ≥ 65 years) during COVID-19 lockdown – results of multiple regression analyses

	Mental wellbeing outcomes														
	Depressive symptoms			Anxiety			Somatization			Global severity index^**a**^			Loneliness		
	*β* coef.	*SE*	*p*	*β* coef.	*SE*	*p*	*β* coef.	*SE*	*p*	*β* coef.	*SE*	*p*	*β* coef.	*SE*	*p*
**Sociodemographic factors**															
Age	.011	.014	.826	.032	.011	.432	.157	.019	**.002**	.091	.036	**.047**	−.063	.010	.231
Female sex (ref. male)	−.009	.138	.798	.046	.145	.205	.002	.195	.949	.015	.382	.667	.110	.106	**.004**
Education (ref. high)															
Low	−.058	.179	.149	−.009	.176	.814	−.011	.274	.802	−.031	.503	.440	−.094	.153	.061
Middle	−.066	.190	.147	−.046	.167	.247	−.043	.259	.336	−.061	.506	.165	−.082	.157	.136
Marital status (ref. married)															
Single	.085	.367	.085	−.058	.291	.142	−.035	.415	.388	−.006	.863	.878	.070	.295	.229
Divorced	.068	.272	.094	−.052	.326	.293	−.018	.572	.774	−.003	1.008	.958	.089	.293	.168
Widowed	.175	.341	**.020**	−.044	.245	.421	.019	.375	.753	.056	.789	.374	.107	.275	.226
Living alone (ref. cohabiting)	.059	.320	.435	.058	.231	.296	.062	.388	.351	.069	.788	.305	−.034	.272	.720
**Aspects of the personal life situation during COVID-19 lockdown**															
Duration of quarantine measures	−.021	.018	.628	−.028	.013	.358	−.047	.022	.208	−.039	.788	.305	−.031	.013	.494
Frequency of direct contact with others over past week	.048	.027	.239	.033	.020	.268	.039	.029	.211	.050	.072	.202	.021	.013	.454
Frequency of indirect contact with others over past week	−.057	.045	.094	−.043	.047	.237	−.033	.069	.382	−.047	.134	.202	.025	.035	.526
Receiving support in daily activities (ref. yes)				.038	.256	.527	−.015	.375	.708	.011	.705	.771	−.020	.165	.587
Partial	.042	.302	.360	.028	.184	.331	−.081	.246	.058	−.027	.470	.507	.020	.122	.637
No	−.005	.174	.905												
Unchanged health services access (ref. yes)															
Partial	.044	.170	.530	.056	.187	.113	.064	.267	.075	.073	.480	**.023**	.006	.127	.855
No	.025	.190	.160	−.033	.166	.339	.046	.262	.241	.025	.494	.499	.057	.155	.224
COVID-19 infection (ref. not applicable)															
Self	.013	.856	.569	.061	1.827	.201	.032	.774	**.028**	.042	3.268	.173	.063	2.024	.411
Household/family member	.042	.599	.273	.007	.426	.811	.053	.645	.085	.051	1.290	.088	.047	.358	.171
Self-isolation (ref. not applicable)	.003	.549	.912	.013	.379	.541	.042	.547	.059	.027	1.040	.211	−.065	.317	**.008**
SelfHousehold/family member	−.003	.378	.936	.000	.349	.988	.018	.367	.453	.006	.768	.812	−.033	.255	.337
**Attitudes towards COVID-19 and associated quarantine measures**															
Being worried	.076	.054	**.038**	.096	.056	**.012**	−.066	.093	.149	.020	.157	.608	.046	.041	.257
Feeling threatened	.020	.071	.676	.046	.063	.283	.056	.102	.257	.058	.191	.216	−.005	.056	.925
Feeling threatened due to age	−.002	.070	.972	−.016	.064	.708	−.004	.091	.933	.000	.175	.999	.071	.053	.177
Feeling threatened due to pre-existing health conditions	.020	.048	.609	−.004	.049	.918	.195	.070	**<.001**	.106	.127	**.004**	−.005	.056	.925
Supportive of the government’s quarantine measures (ref. yes)	.056	.226	.098	.031	.206	.321	.063	.274	**.033**	.065	.577	**.038**	.071	.053	.177
Feeling restricted due to quarantine measures	.108	.050	**<.001**	.061	.050	.066	−.005	.067	.868	.060	.128	**.048**	.002	.037	.956
**Resilience**	−.316	.017	**<.001**	−.347	.016	**<.001**	−.229	.023	**<.001**	−.352	.044	**<.001**	−.146	.013	**<.001**
**Model aspects**															
Constant	3.664	1.184	**.002**	4.090	1.116	**<.001**	1.067	1.528	.485	8.704	3.022	**.004**	5.277	.779	**<.001**
R^2^	.237			.192			.213			.263			.109		

^a^Global severity index: sum of depressive symptoms, anxiety and somatization. Abbreviations: *95%CI* 95% confidence interval; *β coef.* beta coefficient; *p p*-value; *SE* standard error

### Associations of aspects of the personal life situation and mental wellbeing during COVID-19 lockdown

Duration of lockdown was not significantly associated with mental wellbeing. Moreover, frequency of being directly or indirectly in contact with other persons outside of the own household was not significantly associated with any outcome. Partially changed access to health services was associated with overall psychological distress (GSI; *β* = .07; *p* = .023) in reference to unchanged access to health services; however, there was no association with more than partially changed access to health services use (*β* = .03; *p* = .499). Being infected with COVID-19 was associated with increased somatization (*β* = .03; *p* = .028); however, there were only 2 cases in the sample. Having to self-isolate due to exposure to COVID-19 was associated with loneliness (*β* = −.07; *p* = .008).

### Associations of attitudes towards COVID-19 and mental wellbeing during COVID-19 lockdown

Worry about COVID-19 was associated with increased depressive symptoms (*β* = .08; *p* = .038) and anxiety (*β* = .20; *p* = .012), There were no associations with perceived threat by COVID-19 in general or due to age specifically; however, perceived threat by COVID-19 due to pre-existing health conditions was associated with increased somatization (*β* = .20; *p* < .001) and overall psychological distress (GSI; *β* = .11; *p* = .004). Not being supportive of the lockdown was associated with increased somatization (*β* = .06; *p* = .033) and overall psychological distress (GSI; *β* = .07; *p* = .038). Lastly, feeling more restricted by the lockdown was associated with increased depression (*β* = .11; *p* < .001) and overall psychological distress (GSI; *β* = .60; *p* = .048).

### Resilience in regard to mental wellbeing during COVID-19 lockdown

Resilience was strongly associated with better mental wellbeing. Higher resilience indicated less depressive symptoms (*ß* = −.32; *p* < .001), less anxiety (*ß* = −.35; *p* < .001), less somatization (*ß* = −.23; *p* < .001), less overall psychological distress (GSI; *ß* = −.35; *p* < .001), and less loneliness (*ß* = −.15; *p* < .001). Resilience significantly added to the explained variance in all models, which is apparent through comparisons with models that did not control for resilience (see additional Table S1).

## Discussion

We provide results of a representative cross-sectional survey on the mental wellbeing in the old age population (≥ 65 years) during COVID-19 lockdown in Germany with respect to sociodemographic factors, aspects of the personal life situation during lockdown and attitudes towards COVID-19. Results reflect a snapshot after a first lockdown had continuously been in force in Germany for an average of 28 days in April 2020.

Overall, mean scores and prevalence of mental wellbeing outcomes did not differ markedly from those reported by studies undertaken before the pandemic. In detail, normative values for the mean BSI-18 scores [24] measuring depression, anxiety, somatization and overall psychological stress (GSI) in German individuals aged 60–95 years (mean age: 70.8 years) compared to our results were: 2.0 vs. 1.4, 1.6 vs. 1.6, 2.4 vs. 2.2 and 6.0 vs. 5.1, respectively, indicating similar or even lower figures in our sample. There is variability in loneliness measurement, challenging comparisons. However, drawing on pre-pandemic figures from population-based studies, for example, in a random German sample aged 64–94 years using the 12-item version of the UCLA loneliness scale, the frequency of loneliness was 19% in men and 22% in women, respectively [25]. In the cross-national Survey of Health, Ageing and Retirement in Europe (SHARE; age: ≥ 65 years), 13.7% of the German participants stated to feel lonely most of the time [26], and in the European Social Survey, 7% of the Germans aged 60 years and older reported being lonely [27]. As loneliness prevalence in our sample (total: 13.1%; men: 8.8%, women: 16.3%) fell within the range of estimates reported before the pandemic, this did not suggest an increase in loneliness during COVID-19 lockdown; notably though, women’s loneliness was almost as double as high as men’s.

Notably, mean resilience scores (BRS) were slightly above norms for the German old population above 60 years of age. The median BRS score was 3.5, indicating 50% of our study population had this or a lower score, compared to 53.2% or more in the normative population [28]. Sex differences in mean scores and prevalence of mental wellbeing outcomes were typical, with women yielding higher figures than men.

There were hardly any aspects of the personal life situation during COVID-19 lockdown that were associated with mental wellbeing. Experiencing partially changed access to health care services was associated with higher global psychological distress (GSI). This may point to psychological effects of the COVID-19 lockdown in certain subgroups.

On average, older individuals expressed worry about COVID-19, but they were understanding and supportive of the COVID-19 lockdown. There were only few associations of attitudes towards COVID-19 and mental wellbeing. Being worried about COVID-19 was associated with higher depressive symptoms and anxiety. A perceived threat by COVID-19 due to pre-existing health conditions was associated with higher somatization and global psychological distress. Though almost 90% stated to support the lockdown, not being supportive was associated with higher somatization and overall psychological distress; potentially in this group of respondents, individuals were in favor of even stricter quarantine measures to slow the spread of the virus, though the opposite interpretation would be possible as well. In contrast, those feeling more restricted by the COVID-19 lockdown showed higher depressive symptoms. Overall, most life aspects and attitudes did not show associations with mental wellbeing, suggesting that the old age population in Germany was dealing rather well with the crisis 4 weeks into lockdown.

In fact, looking at the results synoptically, we argue that, overall, the mental wellbeing of the German old age population was largely unaltered during COVID-19 lockdown. However, the few significant associations point to a differential impact of the COVID-19 lockdown, implying that certain groups of older individuals may have had more difficulties to adjust to the situation; these could comprise, e.g. individuals with pre-existing health conditions, individuals with certain personality traits, those with small social networks or those with increased medical or care needs [29]. This may indeed require action to think of strategies that help to prevent or attenuate mental health deterioration during the pandemic. Key actions could include low-threshold virtual support groups, psychoeducational outreach or disseminating wellness guides [30]. This requires further investigation and is beyond the scope of this work.

Notably, resilience was strongly associated with all mental wellbeing variables, which explained a large amount of variance and attenuated the associations discussed above. It implies that, on average, older individuals are resilient against disruptive life events, probably because of having mastered crises throughout life. Other studies on the mental wellbeing of older adults during COVID-19 lockdowns draw similar conclusions [5, 31, 32]. E.g., Lopez et al. [31] suggested, based on a study of 60–80 year old Spaniards, the COVID-19 impact may not be as relevant for the older adults’ wellbeing as their appraisals of and resources for managing COVID-19-related problems. Plomecka et al. [5] reported overall lower psychological distress with increasing age, being markedly lower in older age groups, across 12 countries. Thyrian et al. [32] surveyed a convenience sample of older German individuals with cognitive impairment, finding a limited impact of the pandemic on psychological variables including depression, anxiety and loneliness in the short-term. These results, in line with ours, refute the public perception of the “weak and vulnerable older adults”, which has sparked debates and a new rise in ageism over the course of the pandemic [15]. However, these results mostly stem from high-income countries. The impact of COVID-19 lockdowns on the mental and social health of older individuals likely varies considerably between countries, whereby country income level, living conditions, the extent of the outbreak (number of COVID-19 infections, number of deaths), governmental management of the crisis, health care infrastructure and responses of the public health system are important influential factors. A study from the Philippines (a lower-middle income country), for example, concluded that older Filipinos suffered emotionally, spiritually and socially with the country not being sufficiently equipped to manage the crisis [33]. Similar concerns have been expressed from India [34]. This requires differentiated considerations and targeted and tailored response measures in the area of public health.

### Strengths and limitations

A strength of the study is the representative design and the timely data collection period during COVID-19 lockdown in April 2020 in Germany, thereby capturing the immediate impact of the pandemic. However, despite the random multi-stage sampling approach and iterative weighting procedures, results are based on a response rate of 54%. Though such a rate is considered a good response for telephone surveys, it may not entirely rule out deviations in response behavior of those who could not be reached or refused to participate. A limitation is the cross-sectional design, which only allowed for comparisons of outcomes with previous studies. Such comparisons are challenging due to sample deviations or different measurements. However, we were able to draw on normative data for a range of outcomes. Furthermore, due to time restrictions during telephone interviews, we were not able to consider other relevant factors that may be associated with the mental wellbeing of older individuals during the COVID-19 lockdown, for example, specific pre-exiting physical or psychiatric conditions.

## Conclusions

Overall, the mental wellbeing of the old age population in Germany was largely unaltered during COVID-19 lockdown. There was evidence for worry about COVID-19, but in general, the older adults felt socially supported and showed acceptance of and resilience against the challenging pandemic conditions. This does not preclude that certain subgroups are not able to deal well with the crisis and are indeed in need for mental health care. Therefore, further differential analyses as well as longitudinal monitoring of the mental and social health of the older adults over the course of the pandemic and post-pandemic are necessary to provide robust evidence and to understand long-term effects of the COVID-19 pandemic.

## Supplementary Information

**Additional file 1.**

## Data Availability

Data are publicly available at the Figshare repository and can be accessed at 10.6084/m9.figshare.13013657.v1. Data are made available and are shared under the CC BY 4.0 licence.

## Abbreviations

95%CI: 95% confidence interval
ADM: Association of German Market and Social Research Agency’s
BRS: Brief Resilience Scale
BSI-18: Brief Symptom Inventory – 18 items
CASMIN: Comparative Analysis of Social Mobility in Industrial Nations
COVID-19: Corona Virus Disease 2019
GSI: Global Severity Index
SARS-CoV-2: Severe Acute Respiratory Syndrome Coronavirus 2
SD: Standard deviation
UCLA: University of California, Los Angeles
USA: United States of America
WHO: World Health Organization
