# Supplementary material for: Mental wellbeing in the German old age population largely unaltered during COVID-19 lockdown: results of a representative survey
Source: BMC Geriatr. 2020 Nov 23;20:489. doi: 10.1186/s12877-020-01889-x (PMC7681185; doi:10.1186/s12877-020-01889-x)
Supplement: Supplementary file 1 — Additional file 1. [file 12877_2020_1889_MOESM1_ESM.docx]

**Supplemental material**

**Mental wellbeing in the German old age population largely unaltered during COVID-19 lockdown: results of a representative survey**

| **Table S1. Associations of sociodemographic factors, aspects of the personal life situation and attitudes towards COVID-19 with mental wellbeing, without resilience, in the German old age population (n = 1,005, age ≥ 65 years) during COVID-19 lockdown – results of multiple regression analyses.** | | | | | | | | | | | | | | | |
| --- | --- | --- | --- | --- | --- | --- | --- | --- | --- | --- | --- | --- | --- | --- | --- |
|  | **Mental wellbeing outcomes** | | | | | | | | | | | | | | |
|  | **Depressive symptoms** | | | **Anxiety** | | | **Somatization** | | | **Global severity index^£^** | | | **Loneliness** | | |
|  | *β* coef. | *SE* | *p* | *β* coef. | *SE* | *p* | *β* coef. | *SE* | *p* | *β* coef. | *SE* | *p* | *β* coef. | *SE* | *p* |
| **Sociodemographic factors** | | | | | | | | | | | | | | | |
| Age | .045 | .013 | .341 | .077 | .011 | .063 | .183 | .018 | **<.001** | .139 | .035 | .**002** | -.059 | .009 | .230 |
| Female sex (ref. male) | .029 | .140 | .412 | .077 | .151 | **.042** | .020 | .198 | .557 | .046 | .401 | .204 | .133 | .103 | **<.001** |
| Education (ref. high)  Low  Middle | -.036  -.054 | .180  .186 | .384  .228 | -.028  .027 | .166  .180 | .483  .518 | .001  -.036 | .268  .251 | .986  .403 | -.002  -.043 | .511  .493 | .968  .313 | -.089  -.073 | .148  .152 | .069  .167 |
| Marital status (ref. married)  Single  Divorced  Widowed | .093  .060  .152 | .361  .266  .324 | .056  .134  **.035** | -.044  -.055  -.057 | .287  .303  .238 | .264  .235  .281 | -.032  -.032  -.001 | .407  .529  .368 | .425  .582  .991 | .006  -.008  .043 | .855  .932  .756 | .888  .869  .480 | .070  .073  .090 | .284  .284  .264 | .208  .241  .291 |
| Living alone (ref. cohabiting) | .086 | .302 | .231 | .073 | .217 | .162 | .091 | .372 | .155 | .086 | .738 | .176 | -.021 | .260 | .811 |
| **Aspects of the personal life situation during COVID-19 lockdown** | | | | | | | | | | | | | | | |
| Duration of quarantine measures | -.018 | .018 | .671 | -.030 | .013 | .342 | -.050 | .022 | .183 | -.043 | .042 | .233 | -.035 | .013 | .424 |
| Frequency of direct contact with others over past week | .045 | .030 | .306 | .026 | .021 | .414 | .037 | .032 | .273 | .043 | .076 | .286 | .016 | .013 | .562 |
| Frequency of indirect contact with others over past week | -.083 | .045 | **.017** | -.062 | .048 | .091 | -.054 | .068 | .148 | -.069 | .135 | .064 | .014 | .035 | .717 |
| Receiving support in daily activities (ref. yes)  Partial  No | .052  .004 | .296  .173 | .259  .928 | .057  .041 | .265  .187 | .172  .371 | -.001  -.066 | .369  .239 | .983  .116 | .033  -.012 | .735  .479 | .421  .777 | -.030  .003 | .167  .122 | .937  .440 |
| Unchanged health services use (ref. yes)  Partial  No | .055  .014 | .180  .189 | .101  .724 | .068  -.038 | .199  .173 | .069  .291 | .068  .034 | .230  .269 | .058  .385 | .081  .016 | .515  .509 | **.019**  .679 | .015  .051 | .129  .150 | .255  .661 |
| COVID-19 case (ref. no case)  Self  Household/family member | .003  .041 | 1.126  .657 | .920  .330 | .050  .006 | 2.121  .458 | .356  .834 | .025  .053 | 1.037  .665 | .198  .088 | .032  .049 | 4.121  1.468 | .414  .150 | .058  .048 | 2.106  .365 | .465  .162 |
| Self-isolation (ref. no)  Self  Household/family member | -.022  -.020 | .527  .403 | .456  .585 | -.016  -.020 | .378  .343 | .450  .525 | .021  .007 | .579  .390 | .387  .796 | -.005  -.014 | 1.096  .859 | .817  .623 | -.076  -.045 | .284  .250 | **<.001**  .188 |
| **Attitudes towards COVID-19** | | | | | | | | | | | | | | | |
| Being worried | .133 | .054 | **<.001** | .145 | .057 | **<.001** | -.022 | .092 | .622 | .077 | .161 | .055 | .072 | .038 | .065 |
| Feeling threatened | .014 | .074 | .773 | .051 | .067 | .270 | .056 | .103 | .268 | .064 | .200 | .187 | -.016 | .056 | .776 |
| Feeling threatened due to age | .014 | .070 | .768 | -.003 | .067 | .952 | .001 | .090 | .979 | .010 | .180 | .824 | .079 | .053 | .133 |
| Feeling threatened due to pre-existing health conditions | .037 | .050 | .350 | .021 | .052 | .618 | .213 | .071 | **<.001** | .130 | .137 | **<.001** | .003 | .037 | .943 |
| Being supportive of the government’s quarantine measures (ref. yes) | .068 | .241 | .064 | .044 | .225 | .197 | .072 | .283 | **.019** | .078 | .636 | **.025** | .032 | .166 | .378 |
| Feeling restricted due to quarantine measures | .124 | .051 | **<.001** | .089 | .053 | **.012** | .007 | .068 | .821 | .084 | .138 | **.011** | .132 | .035 | **<.001** |
| **Model aspects** | | | | | | | | | | | | | | | |
| Constant | -.944 | .977 | .334 | -1.117 | 1.010 | .269 | -3.517 | 1.307 | **.007** | -6.073 | 2.704 | **.025** | 4.098 | .656 | **<.001** |
| R² | .142 | | | .083 | | | .158 | | | .149 | | | .092 | | |
| Observations | 930 | | | 929 | | | 936 | | | 918 | | | 929 | | |
| **^£^**Global severity index: sum of depressive symptoms, anxiety and somatization.  Abbreviations: 95%CI = 95% confidence interval; *β* coef. = beta coefficient; n/a = not applicable; i.e. independent variable was not considered in the model due to collinearity; OR = odds ratio; *p* = p-value; *SE* = standard error | | | | | | | | | | | | | | | |
